# Efficacy of Naproxen/Fexofenadine (SJP-003) in the Prevention of Side Effects of Influenza Vaccination: Four Case Studies

**DOI:** 10.3390/clinpract12050076

**Published:** 2022-09-09

**Authors:** Pantea Kiani, Thomas A. Dahl, Jacqueline M. Iversen, Andrew Scholey, Joris C. Verster

**Affiliations:** 1 Division of Pharmacology, Utrecht Institute for Pharmaceutical Sciences (UIPS), Utrecht University, 3584CG Utrecht, The Netherlands; 2 Sen-Jam Pharmaceutical, 223 Wall St., #130, Huntington, NY 11743, USA; 3 Nutrition Dietetics and Food, School of Clinical Sciences, Monash University, Melbourne, VIC 3168, Australia; 4 Centre for Human Psychopharmacology, Swinburne University, Melbourne, VIC 3122, Australia

**Keywords:** influenza, vaccination, side effects, pain at injection site, flu-like symptoms, naproxen, fexofenadine, SJP-003, willingness to vaccinate

## Abstract

The influenza virus is associated with sickness, and in particular among vulnerable populations such as elderly and those with underlying disease with hospitalization and increased mortality rates. Vaccination is an effective way to prevent infection with influenza. However, undesirable side effects of the vaccination are commonly experienced, and comprise one of the primary reasons for a substantial group of individuals to refrain from vaccination. An effective treatment against vaccination side effects could increase the overall willingness to vaccinate against influenza. Here, four cases are presented that self-administered SJP-003 (a combination of 220 mg naproxen sodium, directly followed by a single oral dose of 60 mg fexofenadine HCL), 2 h before and 10 h after influenza vaccination. No flu-like symptoms and pain at the injection site were reported. These observations warrant further investigation of SJP-003 in double-blind, placebo-controlled clinical trials.

## 1. Introduction

Many individuals now and then experience a short sickness period due to influenza. Although, for them, influenza does not require hospitalization, it may have a significant impact on job performance and daily activities [1]. In addition, the US Centers for Disease Control and Prevention (CDC) reported that, from 1 October 2021 through 21 May 2022 there have been about 74,000–150,000 hospitalizations due to influenza [2], and influenza has been related to increased mortality rates [2]. As such, influenza is a yearly returning health risk, in particular among vulnerable populations such as elderly and individuals with underlying disease [3].

The most effective method of preventing influenza is vaccination [4,5,6]. Studies have shown that vaccination prevents between 40–60% of influenza cases, and significantly reduces the severity of influenza symptoms once infected [7,8,9]. However, a substantial number of individuals are discouraged from obtaining vaccinations due to a variety of mild to moderate undesirable side effects, which typically appear shortly after a vaccination. For example, the package insert of the Fluzone^®^ (Sanofi Pasteur, Lyon, France) quadrivalent influenza vaccine (Sanofi Pasteur) reports considerable percentages of people reporting side effects [10]. These side effects, either local or systemic, were observed in studies conducted in both adults [11] and elderly [12]. Most frequently reported side effects in adults and elderly were pain at the injection side (47.4% and 32.6%, respectively), myalgia (23.7% and 18.3%, respectively), headache (15.8% and 13.4%, respectively), and malaise (10.5% and 10.7%, respectively). It can be assumed that some of these vaccination side effects may negatively impact mood and performance of daily activities.

Although most side effects disappear within a couple of days after vaccination, the fear of having adverse reactions due to vaccinations may discourage individuals from obtaining vaccinations. Indeed, European and US studies found that fear of side effects was an important motive to refrain from getting vaccinated [13].

Give this, the availability of effective and safe medicines that prevent undesirable side effects of vaccination would be an important asset to reduce resistance against vaccination. However, at present, there are no medicines available to prevent vaccination side effects. In the US, the Immunization Action Coalition (IAC) advises vaccinated people who experience side effects of vaccination to apply a cold compress to the injection site to ease local side effects such as pain and swelling [14]. Vaccinated individuals could also consider taking an OTC pain reliever to reduce pain at the injection site or headache, or an antipruritic drug to reduce itch.

Instead of symptom management after vaccination, it would be more favorable if undesirable side effects could be prevented. With this aim in mind, a new drug, SJP-003 (a combination product of the NSAID naproxen and the antihistamine drug fexofenadine), is currently being developed. SJP-003 aims to prevent or reduce side effects of vaccination and/or shorten their duration [15]. Here, we present four case studies that self-administered naproxen and fexofenadine with the aim to prevent possible side effects after vaccination against influenza.

## 2. Materials and Methods

Four individuals had learned about SJP-003 via the Internet and contacted Sen-Jam Pharmaceutical about the availability of the product. They were informed that SJP-003 was not marketed yet, but that naproxen and fexofenadine are freely available over the counter in the USA. Each of the four individuals was scheduled for a vaccination against influenza. They purchased the drugs themselves and were asked to report the presence and severity of any vaccination side effects to the corresponding author.

No ethics approval was needed for the self-administration of these existing approved drugs. The four individuals were advised to self-administer a single oral dose of 220 mg naproxen sodium, directly followed by a single oral dose of 60 mg fexofenadine HCL. They were informed that the recommended dosing schedule was to self-administer the drug combination 1–2 h before receiving the intramuscular (deltoid) injection of 0.5 mL of the Fluzone^®^ quadrivalent influenza vaccine (Sanofi Pasteur, Lyon, France), followed by a second dosing of naproxen and fexofenadine 8–10 h after vaccination.

They were asked to rate the severity of the side effects of the vaccination on a scale ranging from 0 (absent) to 10 (extreme). The side effects included flu-like symptoms, including fever, headache, chills, and muscle aches. In addition, pain at the injection site was rated using the same scale. The side effects were rated 2 h and 10 h after vaccination. Side effect ratings were reported via phone or text messages to the corresponding author.

## 3. Results

An overview of the four cases is given in Table 1. All four individuals self-administered the treatment approximately 2 h before the influenza vaccination. They all received their vaccine injection to the left deltoid muscle. A second dose of naproxen and fexofenadine was self-administered 10 h after vaccination by all individuals, except case 3. All four individuals were healthy by self-report, without underlying diseases.

No flu-like side effects of vaccination were reported by any of the individuals. At 2 h after vaccination, they reported pain at the injection site score of 1. No other side effects were reported. At 10 h after vaccination, no side effects were reported.

## 4. Discussion

The four cases described here suggest that SJP-003 is effective in preventing or reducing side effects after a vaccination against influenza. However, before making any causal conclusion on the efficacy of SJP-003, a case–control study should be conducted to provide further evidence. The observations do warrant further investigation of SJP-003 in a double-blind, placebo-controlled clinical trial including both cases and controls. Although the current four cases were individuals that were scheduled for a influenza vaccination, no medical history or demographics other than age and sex were collected. Future studies should have clear inclusion and exclusion criteria, record medical history, and concurrent medication use. Such a clinical trial should further investigate the efficacy of SJP-003 in preventing influenza vaccination side effects.

Whereas naproxen is known for its anti-inflammatory properties [16], fexofenadine is most frequently used for its antihistaminergic properties [17]. However, fexofenadine also exhibits anti-inflammatory properties [18]. It is therefore hypothesized that SJP-003 has an additive or synergistic effect in preventing or reducing pain and flu-like (immune-related) vaccination side effects. Previous research has suggested that administering NSAIDS alone may reduce vaccine efficacy [19]. However, various other studies presented inconsistent results, and it therefore remains to be determined if any clinically relevant effects of NSAIDs on vaccine efficacy exist [20]. Therefore, future clinical trials should also evaluate if administering a combination of naproxen and fexofenadine will affect vaccine efficacy. Thus, more research is needed to elucidate the mechanistic pathways that may explain the efficacy and safety of SJP-003. Finally, future research should also investigate whether SJP-003 is effective in preventing side effects of other types of vaccinations, including vaccination against coronavirus disease 2019 (COVID-19) [21]. A recent case study was published suggesting that SJP-003 was effective in reducing side effects after multiple travel vaccinations [22].

In conclusion, the combination of (1) the outcome of the case reports presented here, (2) the wish to prevent side effects of vaccinations, (3) the necessity to address fear of vaccination, and (4) the need to increase the willingness to vaccinate among the general population, support that further research on the efficacy and safety of SJP-003 is warranted.

## Figures and Tables

**Table 1:** Characteristics of the four cases.

			Self-Administered Treatment	
	Sex	Age	2 h Prior to Vaccination	10 h after Vaccination
Case 1	Female	53	Yes	Yes
Case 2	Female	59	Yes	Yes
Case 3	Male	53	Yes	No
Case 4	Male	29	Yes	Yes

## Data Availability

All available data are presented in this article.
